# Specific plasticity of the anemone *Anthopleura hermaphroditica* to intertidal and subtidal environmental conditions of the Quempillén estuary

**DOI:** 10.1371/journal.pone.0279482

**Published:** 2023-01-05

**Authors:** Víctor M. Cubillos, Felipe E. Ramírez, Daniela A. Mardones-Toledo, Nelson Valdivia, Oscar R. Chaparro, Jaime A. Montory, Edgardo A. Cruces

**Affiliations:** 1 Instituto de Ciencias Marinas y Limnológicas, Facultad de Ciencias, Universidad Austral de Chile, Valdivia, Chile; 2 Laboratorio Costero de Recursos Acuáticos de Calfuco, Facultad de Ciencias, Universidad Austral de Chile, Valdivia, Chile; 3 Centro FONDAP de Investigación Dinámica de Ecosistemas Marinos de Altas Latitudes (IDEAL), Facultad de Ciencias, Universidad Austral de Chile, Valdivia, Chile; 4 Centro i~mar, Universidad de Los Lagos, Puerto Montt, Chile; 5 Centro de Investigaciones Costeras, Universidad de Atacama (CIC-UDA), Copiapó, Chile; KAUST University, SAUDI ARABIA

## Abstract

The cellular capacity of marine organisms to address rapid fluctuations in environmental conditions is decisive, especially when their bathymetric distribution encompasses intertidal and subtidal zones of estuarine systems. To understand how the bathymetric distribution determines the oxidative damage and antioxidant response of the estuarine anemone *Anthopleura hermaphroditica*, individuals were collected from upper intertidal and shallow subtidal zones of Quempillén River estuary (Chile), and their response analysed in a fully orthogonal, multifactorial laboratory experiment. The organisms were exposed to the effects of temperature (10°C and 30°C), salinity (10 ppt and 30 ppt) and radiation (PAR, > 400–700 nm; PAR+UV-A, > 320–700 nm; PAR+UV-A+UV-B, > 280–700 nm), and their levels of lipid peroxidation, protein carbonyl and total antioxidant capacity were determined. The results indicated that the intertidal individuals of *A*. *hermaphroditica* presented higher levels of tolerance to the stressful ranges of temperature, salinity, and radiation than individuals from the subtidal zone, which was evident from their lower levels of oxidative damage to lipids and proteins. These results were consistent with increased levels of total antioxidant capacity observed in subtidal organisms. Thus intertidal individuals could have greater plasticity to environmental variations than subtidal individuals. Future studies are needed to understand the mechanisms underlying stress adaptation in individuals from this estuarine anemone subjected to different environmental stressors during their life cycles.

## Introduction

The intertidal zone is an area in which organisms are exposed to successive periods of exposure and immersion. This area is one of the harshest environments for the survival of sessile organisms, since they are exposed daily to highly stressful situations as a result of the rapid changes in the surrounding environmental conditions (e.g. tidal fluctuations) [1, 2]. The above generates patterns of distribution and abundance through a bathymetric gradient from intertidal to subtidal environments [3, 4]. Intertidal organisms face wide environmental variability and long periods of aerial exposure, developing higher physiological tolerance limits than subtidal organisms [5–7]. Some populations of marine invertebrates can live in both intertidal and subtidal zones [8–10], however, they may present physiological differences in their response to environmental fluctuations within the same species [11, 12].

Rapid changes in the surrounding environment of a given habitat will allow only some species to be able to colonize such environments successfully [13, 14], due to their efficiency in quickly adjusting their physiology to the new environmental conditions [15]. Exposure of animals to environmental stress levels above physiological tolerance limits can exacerbate the production of reactive oxygen species (ROS) [16, 17]. When ROS levels exceed the antioxidant capacity to intercept and neutralize these cytotoxic compounds, changes are generated in the structure and functionality of lipids, proteins and DNA [16, 18]. This process, known as oxidative stress [19], can lead to an imbalance in cellular homeostasis [18]. Organisms living in intertidal habitats are exposed to higher ranges of stress than those living in subtidal habitats where abiotic environmental conditions are more stable [20, 21]. Organisms in both environments develop both behavioural and physiological strategies that cope with fluctuations in the abiotic stressors associated with the bathymetric gradient [2, 22].

*Anthopleura hermaphroditica* is a small symbiont anemone which has a wide latitudinal distribution along the Chilean coast (23° S– 41° S), inhabiting bays and estuaries in densities of up to 10,000 individuals m^-2^ [23, 24]. In southern Chile, a population of *A*. *hermaphroditica* inhabits the intertidal and subtidal areas of the Quempillén River estuary (Ancud, Chiloé Island, Chile). This mid-latitude estuary in the Southern Hemisphere has high levels of ultraviolet B radiation (UV-B; > 280–320 nm), which can reach up to 50% more than those observed at equivalent latitudes in the Northern Hemisphere [25].

The temperature of the water column of the Quempillén estuary remains close to 10°C during winter, while during summer it can fluctuate between 14°C and 31°C [26]. Due to tidal fluctuations and constant rainfall in the area, the salinity of the estuary can decreases from 30 ppt to 9 ppt in a period of 6 hours approximately [27]. These low salinity levels can last for periods of up to 72 h, which forces individuals to isolate and close up as a mechanism to minimize osmotic damage in their soft tissues [22, 27]. A previous study showed that wide changes in UV-B levels, temperature and salinity throughout the year in the Quempillén estuary were related to increased levels of lipid peroxidation and protein carbonyl in *A*. *hermaphroditica* by 4 and 20-fold, respectively, between anemones acclimated to summer and winter [26].

Here we test the hypothesis that, since the population of *A*. *hermaphroditica* covers the estuary of the Quempillén River from upper intertidal zones to shallow (ca. 1 m depth) subtidal zones, the impact of environmental stressors can have different cellular responses on *A*. *hermaphroditica* individuals collected from both extremes of the species’ vertical distribution. Thus, this study aims to evaluate the specific plasticity of *A*. *hermaphroditica* to intertidal and subtidal conditions, and the oxidative response of adults exposed to a combination of salinity, temperature, UV-B, and exposure time.

## Material and methods

### Sampling site

**Fig 1:**
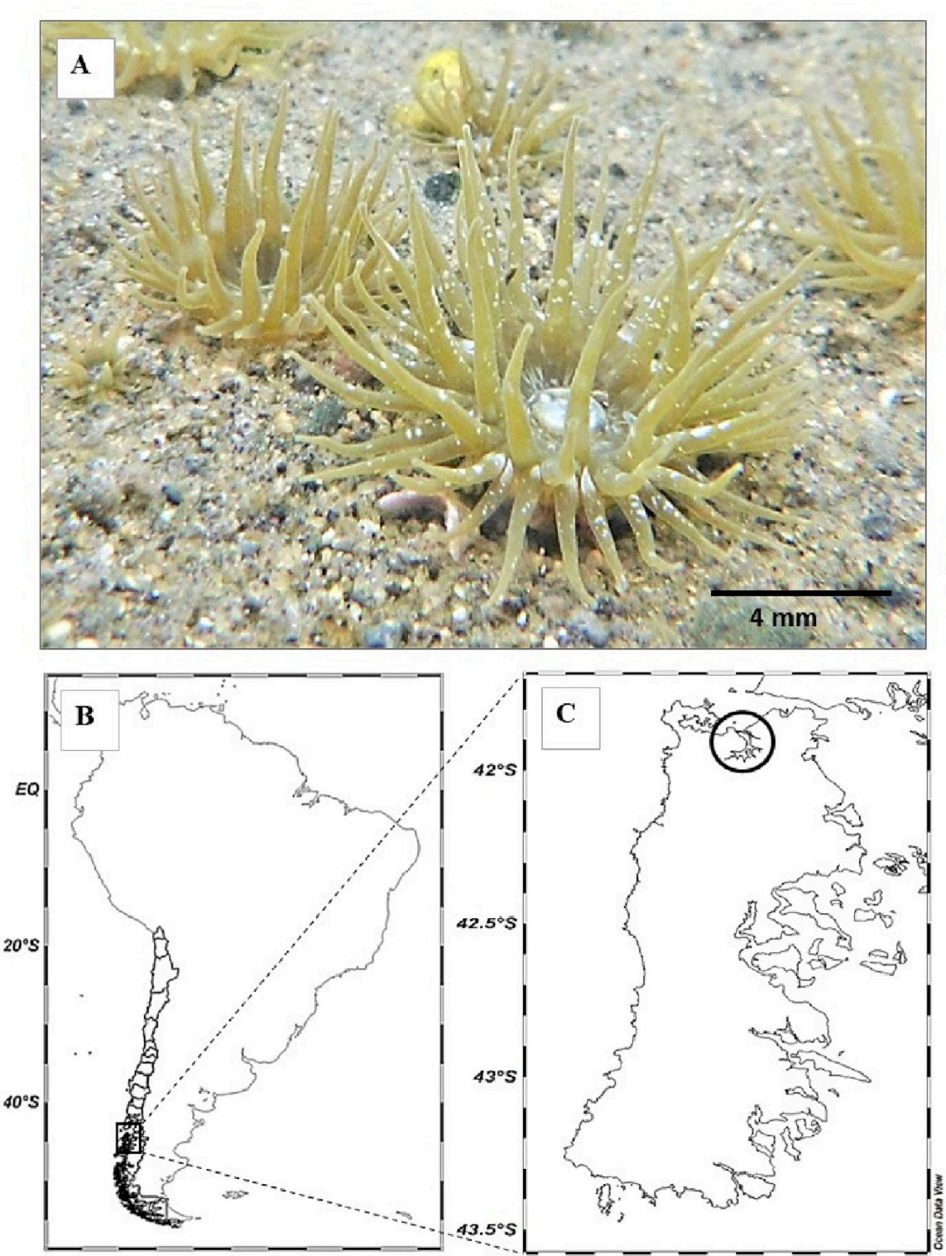
*Anthopleura hermaphroditica*. Adults of the estuarine anemone *A*. *hermaphroditica* (A) collected from southern Chile (Chiloé Island) (B) in the intertidal and subtidal areas of the Quempillén estuary (C) (41°520S; 73°460W).

Adult individuals of *A*. *hermaphroditica* were collected during summer, 2019 from the intertidal and shallow subtidal (ca. 1 m depth) zones of the Quempillén River estuary (41° 52 ’S; 73° 46’ W Ancud, Chiloé, Fig 1), and immediately transferred to the Coastal Laboratory of Aquatic Resources of Calfuco (Universidad Austral de Chile).

Experimental anemones were kept in aquaria with seawater (30 ppt) for a period of three days prior to carrying out the experiment. During that period, seawater temperature was regulated (14–15°C) by using a water chiller system, and oxygen was continuously provided by an air pump fitted with a bubbling stone. The photoperiod was defined as 12:12 h (light: dark) and the illumination was achieved by using photosynthetic active radiation (PAR, > 400–700 nm). Light was provided by a fluorescent tube lamp (Phillips Daylight 40 W) placed 30 cm above the acclimation aquaria, generating an irradiance of 100 μmol m^-2^ s^-1^.

### Experimental design and setup

A total of 288 individuals of *A*. *hermaphroditica* (approximately 3.5 mm basal disc diameter) were used to evaluate how bathymetric location (intertidal and subtidal) influence the cellular responses (oxidative response and total antioxidant capacity) of this estuarine anemone when exposed to different levels of salinity (10 and 30 ppt), temperature (10°C and 30°C), and radiation (P = PAR, PA = PAR+UV-A and PAB = PAR+UV-A+UV-B) over three time periods (0, 24, and 48 h). Random groups of estuarine anemones were sampled at each sample time. Thus, our experimental design was fully orthogonal; that is, each anemone was exposed to a single combination of the four experimental factors and time (72 unique combinations in total; n = 4 individuals per combination). This design allowed us to assess the separate and interactive effects of variations in bathymetric origin, salinity, temperature, radiation and exposure time on estuarine anemone oxidative damage and antioxidant capacity.

**Fig 2:**
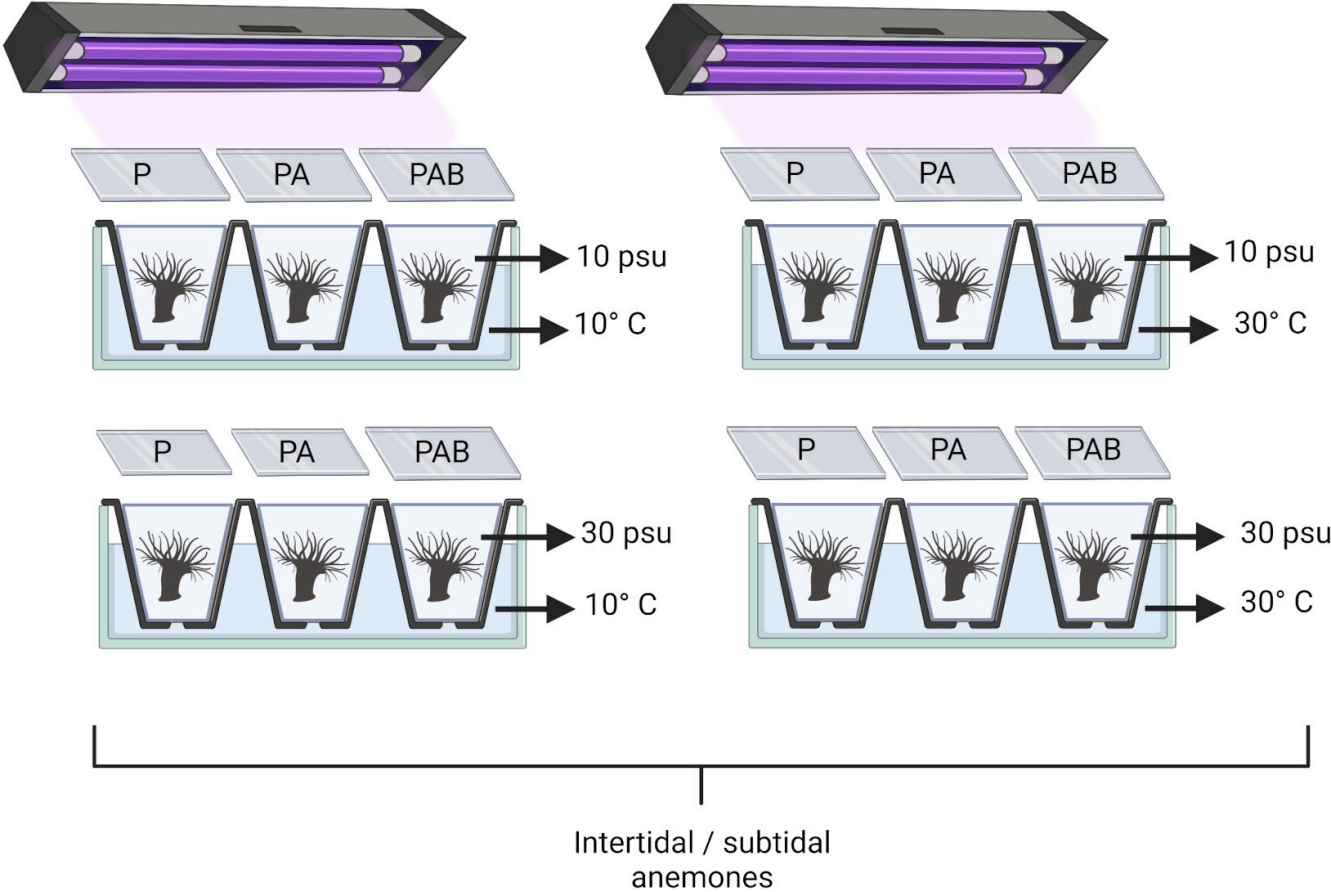
*Anthopleura hermaphroditica*. Experimental setup of the multifactorial experiment where intertidal and subtidal anemones of *A*. *hermaphroditica* were exposed to a combination of temperatures (10°C and 30°C), salinity (10 and 30 ppt), and radiation (P: PAR, PA: PAR+UV-A, and PAB: PAR+UV-A+UV-B) for 0, 24 and 48 h. Diagram was created using BioRender (BioRender.com).

Animals were individually placed in small plastic aquariums (4 x 3 x 3 cm) containing 10 ml of filtered sea water (0.5 μm) and assigned to the experimental combinations of salinity, temperature, and radiation over three time periods (indicated above). Subsequently, the aquaria were placed in a water bath at temperatures of 10°C or 30°C. Radiation treatments were generated using a combination of PAR fluorescent tubes (Phillips Daylight 40 W / 100 μmol m^-2^ s^-1^), UV-A (Phillips TL40W / 03 40 W / 62 W m^-2^), and UV-B (20 W / 2.4 W m^-2^ Phillips UV-B TL2012). The fluorescent tubes were placed 30 cm above the experimental aquaria. P (> 400–700 nm), PA (> 320–700 nm) and PAB (> 280–700 nm) radiation treatments were generated with Clear-220 PAR (Johnson, UK), Folanorm-AFS (Folex, Germany), and Cellulose acetate (CDA; Graphix / USA) cut-off filters, respectively (Fig 2).

### Oxidative damage and antioxidant capacity

Estuarine anemones were sampled at the beginning of the experiment (“time 0 h”) and every 24 h until the completion of the experiment at 48 h. Water was drained from the gastrovascular cavity of each anemone by pressing it with a soft tissue. Then they were transferred to a centrifuge tube (1.5 ml) and included in liquid nitrogen for 10 s before being stored in an ultra-freezer (-80°C) until determination of cellular damage (lipid peroxidation and protein carbonyl) and total antioxidant capacity.

To avoid thawing of estuarine anemone tissue during the grinding process, a ceramic mortar and pestle were immersed in liquid nitrogen. Then frozen tissue was ground manually to a fine powder. Finally, the frozen powder was quickly weighted (according to each biochemical analysis) in a 1.5 ml centrifuge tube using a frozen metallic spatula. Loaded tubes were stored in an ultra-freezer (-80°C) until biochemical analyses were performed.

Lipid peroxidation levels were determined by quantifying malondialdehyde (MDA) using the TBARS method [28], which was modified for microplates [26]. For this, 30 mg of pulverized frozen tissue was homogenized with 500 μl TCA (0.1% w/v) and centrifuged at 4°C for 10 min at 13,000 RPM. Then a200 μl aliquot of the supernatant was homogenized with 500 μl of a solution or TCA (20% w/v) and TBA (0.5% w/v), and then heated at 80°C for 30 min using a Thermomixer (Eppendorf, Thermomixer, Hauppauge, NY).

Samples were immediately placed on ice for 5 min, and then centrifuged at 13,000 rpm for 5 min at 4°C. Finally, the absorbance of the supernatant was determined at 520 nm using a Zenyth 200 microplate reader (Anthos, Biochrom Ltd., Cambridge, UK). Lipid peroxidation levels were expressed in nmol MDA g FW^-1^.

Protein oxidation levels were estimated by quantifying protein carbonyl, which was determined using the DNPH (dinitrophenylhydrazine) protocol [29]. For this, 80 mg of frozen tissue previously ground with liquid nitrogen was homogenized with saline extraction buffer (1% polyvinylpyrrolidone and 0.1 mM EDTA) and centrifuged at 13,500 RPM at 4°C for 20 min.

Proteins from the extract were precipitated with 200 μl TCA 20%, frozen for 30 min at -20°C, then separated from the homogenate by centrifugation (13,500 rpm for 10 min at 4°C). The supernatant was discarded, and the pellet was re-suspended with 300 μl of 100 mM DNPH in 2N HCl and incubated at room temperature for one hour. Then 500 μl of TCA 20% was added, and frozen again for 15 min at -20°C. The solution was centrifuged at 13,500 rpm for 10 min, and the supernatant was eliminated. The pellet was re-suspended with a solution of ethanol: ethyl acetate (1:1) and then centrifuged at 14,000 rpm for 10 min at 4°C. The supernatant was discarded again, and the resulting pellet was re-suspended with guanidine HCl 6M and centrifuged at 14,000 for 20 min at 4°C. Finally, the absorbance of the supernatant was determined at 380 nm using a plate reader.

A soluble protein extract was generated to begin the determination of protein carbonyls, for which 1 ml of 1X PBS buffer pH 7.4 100 mM EDTA and 1% PVPP was added to 100 mg of frozen ground tissue and centrifuged at 14,000 rpm for 20 min. The total concentration of the protein extract was determined according to the specifications of the commercial protein determination kit (Pierce BCA). After the concentrations were obtained, the concentration of all samples was equalized to a minimum of 200 μg protein per mg frozen weight. Then an aliquot of 200 μl of protein extract was used to determine carbonyl according to the protocol of DNPH [29]. Protein carbonyl levels were expressed in μmol carbonyl mg protein^-1^.

Levels of total antioxidant capacity of *A*. *hermaphroditica* were determined by homogenizing 60 mg of frozen tissue, previously ground with liquid nitrogen, in 1 ml of 70% acetone, mixing well using a vortex and sonicating in an ultrasonic bath for 2 h at 4°C. Then samples were centrifuged for 10 min at 7,500 rpm (4°C). The supernatant was reserved in a new tube and placed under a laboratory fume hood to evaporate acetone to final volume of 500 μl, which facilitated the estimation of total antioxidant capacity by the extraction of phenolic compounds.

The antioxidant capacity of phenolic compounds was determined using the protocol of 2,2-diphenyl-1-picrylhydrazyl (DPPH) [30, 31]. A 200 μl volume of DPPH (150 μM in 70% acetone) was placed in each well of a 96-well microplate, and 25 μl of the extract of each sample was added, then the absorbance was determined at 520 nm through a kinetic loop for 2 h (37°C) using a Zenith 200 microplate reader (Anthos, Biochrom Ltd., Cambridge, UK). Finally, levels of total antioxidant capacity were estimated using Trolox (synthetic antioxidant / 6-Hydroxy-2,5,7,8-tetramethylchromane-2-carboxylic acid) as a reference, expressing the values in mg Trolox Eq g FW^-1^.

### Statistical analysis

General linear models were used to analyse the lipid peroxidation, protein carbonyl and total antioxidant capacity production data separately. For each response variable, the global model took the form of *y* = Z*(*T*+*R*+*Te*+*S*), where *y* is the predicted response variable and *Z*, *T*, *R*, *Te*, and *S* are zone (two levels: intertidal or subtidal), time (three levels: 0, 24, or 48 h), radiation (three levels: P, PA, or PAB), temperature (two levels: 10 or 30°C) and salinity (two levels: 10 or 30 ppt), respectively. Plots of residual *vs*. fitted values and expected *vs*. observed quantiles (Q-Q plots) were used as model diagnostics. ANOVA tables were computed for each model. Pairwise comparisons were used for each statistically significant term (P < 0.05) in the ANOVA table. When main effects were significant, the marginal means were compared; in the case of interactive effects, the marginal means of the experimental treatments were compared separately for each zone (intertidal or subtidal). Main effects of factors involved in significant interactions were not analysed. P-values were adjusted with the Tukey method for factors with three levels (time and radiation) to control Type-I error rate. Statistical analyses were carried out using R statistical package [32].

### Ethics statement

The estuarine anemone *Anthopleura hermaphroditica* is not an endangered or protected species. However, all required permissions to sample, remove or use individuals for experimental procedures were approved by the Ethics Committee of the Universidad Austral de Chile. Certificate number 356/2019.

## Results

### Lipid peroxidation levels

**Fig 3:**
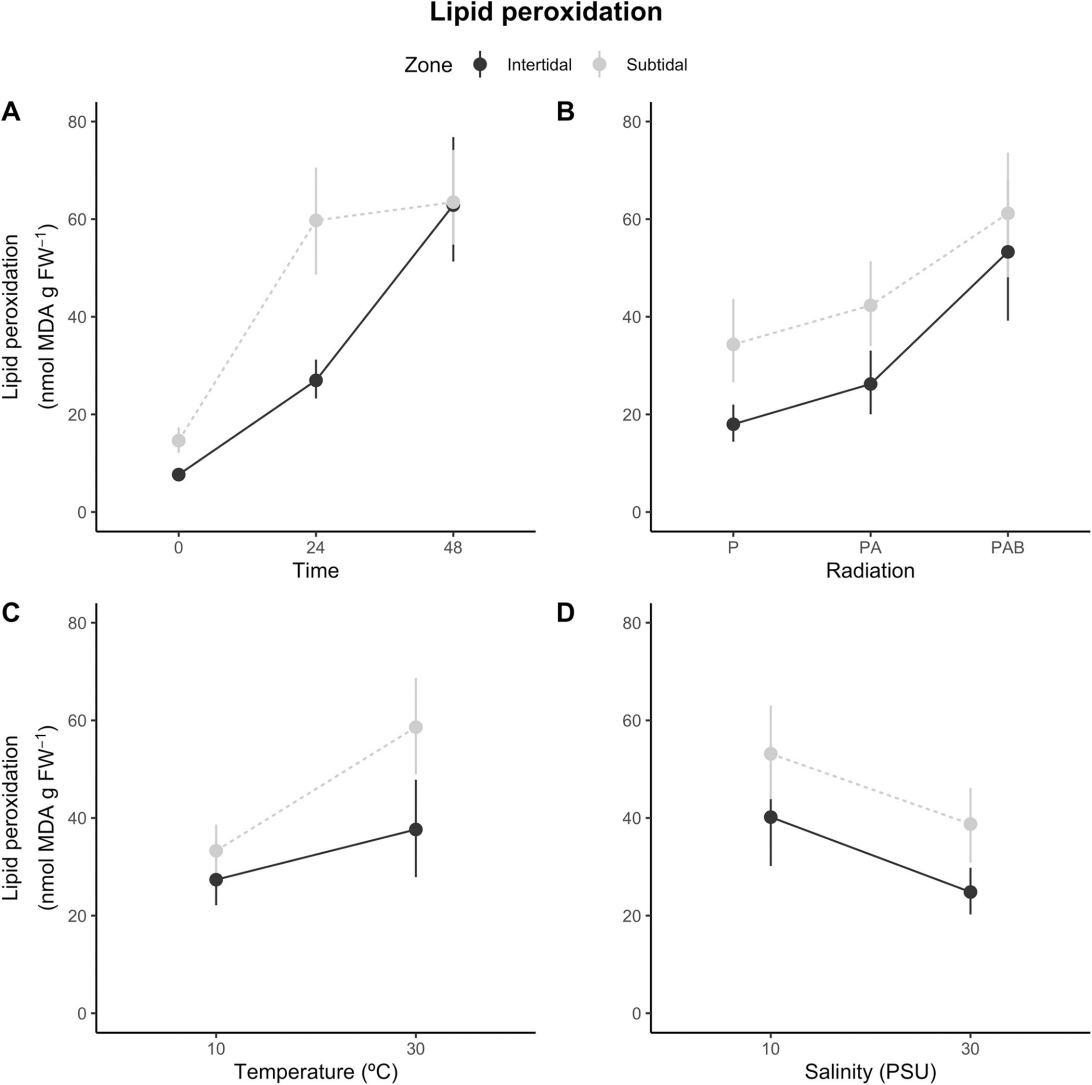
*Anthopleura hermaphroditica*. Lipid peroxidation: zone-dependent responses of estuarine anemones to variations in (A) exposure time, (B) radiation, (C) temperature and (D) salinity treatments. Values are provided as means ± confidence intervals (n = 4).

The temporal changes of lipid peroxidation in *A*. *hermaphroditica* were dependent on individuals’ distribution either in the intertidal or subtidal zone (Fig 3), which was supported by a significant zone X time interaction effect on lipid peroxidation (Table 1).

**Table 1:** *Anthopleura hermaphroditica*.

**Lipid peroxidation**			
*R*^*2*^ *= 0*.*62*			
	df	F	P
Zone (Z)	1	22.7	**<0.001**
Time (T)	2	115.6	**<0.001**
Radiation (R)	2	43.6	**<0.001**
Temperature (Te)	1	39.8	**<0.001**
Salinity (S)	1	27.8	**<0.001**
Z x T	2	12.2	**<0.001**
Z x R	2	1.0	0.380
Z x Te	1	7.1	**0.008**
Z x S	1	0.0	0.865
Residuals	274		
**Protein carbonyls**			
*R*^*2*^ *= 0*.*75*			
	**df**	**F**	**P**
**Z**	1	1.2	0.271
**T**	2	340.9	**<0.001**
**R**	2	8.4	**<0.001**
**Te**	1	6.6	**0.011**
**S**	1	0.5	0.464
**Z x T**	2	55.4	**<0.001**
**Z x R**	2	1.7	0.188
**Z x Te**	1	9.3	**0.003**
**Z x S**	1	0.2	0.662
**Residuals**	274		
**Total antioxidant capacity**			
*R*^*2*^ *= 0*.*71*			
	**df**	**F**	**P**
**Z**	1	309.7	**<0.001**
**T**	2	97	**<0.001**
**R**	2	6.1	**0.003**
**Te**	1	4.9	**0.028**
**S**	1	0.1	0.790
**Z x T**	2	60.2	**<0.001**
**Z x R**	2	2.5	0.081
**Z x Te**	1	4.9	**0.027**
**Z x S**	1	9.0	**0.003**
**Residuals**	274		

Summary of statistical analyses of the effects of bathymetric zone (intertidal or subtidal), time (0, 24, or 48 h), radiation (P, PA, or PAB), temperature (10 or 30°C), and salinity (10 or 30 ppt) treatments on peroxidation, protein carbonyl levels and total antioxidant capacity of the estuarine anemone *A*. *hermaphroditica*. For each response variable, the global model took the form of *y* = Z*(*T*+*R*+*Te*+*S*). Goodness-of-fit (R^2^), degrees of freedom (df), and P-values are provided. P-values < 0.05 are in bold.

According to the post-hoc analyses, peroxidation significantly increased between exposure times in the intertidal anemones, while for the subtidal anemones it increased between 0 and 24 h from exposure to experimental treatments, but not between 24 and 48 h (S1 Table). In contrast, the effect of radiation on peroxidation was independent of zone (Fig 3; Table 1). Lipid peroxidation levels remained similar between P- and PA-exposed estuarine anemones across both zones, but was significantly higher in the PAB-exposed anemones compared to the other two groups (Table 1). The increase of temperature from 10 to 30°C triggered zone-dependent estuarine anemone responses (Fig 3; Table 1). Lipid peroxidation in the intertidal estuarine anemones increased 10.3 nmol MDA g FW^-1^ between the 10 and 30°C groups. This increase was 2.5-fold greater than for the intertidal organisms than for subtidal estuarine anemones (S1 Table). Finally, the reduction in salinity from 30 to 10 ppt caused a significant and zone-independent increase in lipid peroxidation levels (Fig 3; Table 1). The statistical model accounted for 62% of the variation in lipid peroxidation (R^2^ = 0.62).

### Protein carbonyl levels

**Fig 4:**
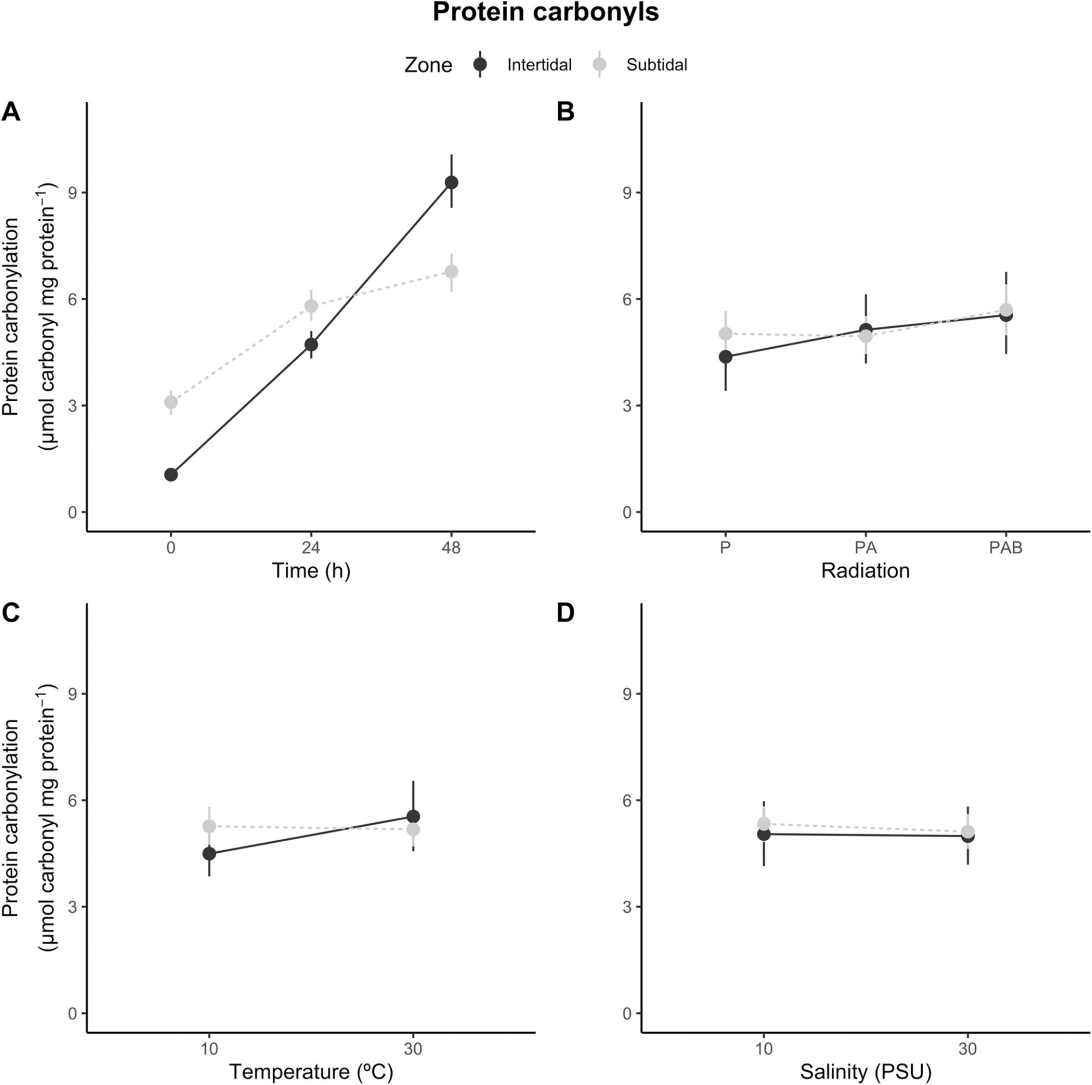
*Anthopleura hermaphroditica*. Protein carbonyl levels: zone-dependent responses of the estuarine anemone *A*. *hermaphroditica* to variations in (A) exposure time, (B) radiation treatment, (C) temperature and (D) salinity treatments. Values are provided as means ± confidence intervals (n = 4).

The increase in protein carbonyl levels between consecutive exposure times was dependent on the bathymetric distribution zone (Fig 4A; Table 1).

Protein carbonyl levels increased between consecutive exposure times at a similar rate in the intertidal estuarine anemones (3.7 and 4.6 μmol carbonyl mg protein^-1^ for the 0–24 and 24–48 h comparisons, respectively). For the subtidal estuarine anemones there was an increase of 2.71 μmol carbonyl mg protein^-1^ between 0 and 24 h, then an increase of 1.0 nmol μmol carbonyl mg protein^-1^ between 24 and 48 h (S1 Table). The effect of radiation exposure on protein carbonyl levels was similar for both bathymetric zones (Fig 4B). However, the interaction between time and radiation showed a non-significant effect (Table 1). The P-PA contrast was not significant for this response variable, while both P-PAB and PA-PAB showed significant increases (S1 Table). The increase in temperature generated an increase in carbonylation only in the intertidal habitat (Fig 4C; Table 1; S1 Table). Finally, neither the main nor the interaction effect involving salinity influenced the generation of protein carbonyl levels in the experimental estuarine anemones (Fig 4C; Table 1). The global model explained 75% of the variation in protein carbonyls (R^2^ = 0.75).

### Total antioxidant capacity

**Fig 5:**
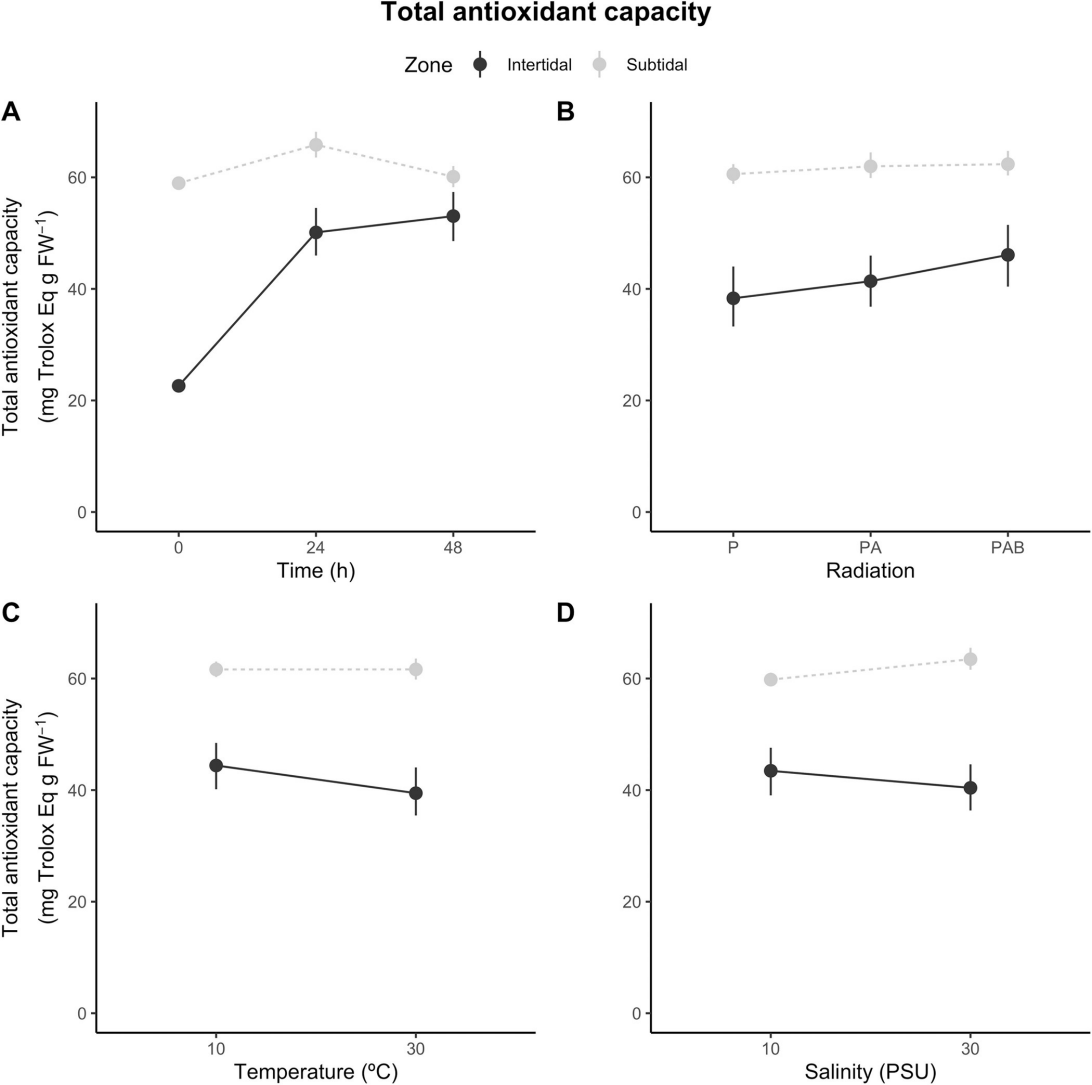
*Anthopleura hermaphroditica*. Total antioxidant capacity: zone-dependent responses of the estuarine anemone *A*. *hermaphroditica* to variations in (A) exposure time, (B) radiation treatment, (C) temperature and (D) salinity treatments. Values are provided as means ± confidence intervals (n = 4).

On average, total antioxidant capacity was larger in the subtidal than the intertidal estuarine anemones (Fig 5).

Nevertheless, experimental factors mediated the spatial pattern of this process. A strong interdependent effect of exposure time and zone on total antioxidant capacity was observed (Fig 5A; Table 1). The total antioxidant capacity of the intertidal estuarine anemones increased greatly between 0 and 24 h (27.5 mg Trolox Eq. g FW^-1^), and then remained invariable between 24 and 48 h (Table 1). For the subtidal estuarine anemones, total antioxidant capacity increased to 6.9 mg Trolox Eq. g FW^-1^ from 0 to 24 h, and then decreased to 5.7 mg Trolox Eq. g FW^-1^ from 24 to 48 h (S1 Table). The differences between radiation treatments appeared to be larger in the intertidal than the subtidal estuarine anemones (Fig 5B). However, this pattern was not supported by the statistical model (P = 0.08 for the radiation by zone interaction; Table 1). The PAB radiation treatment triggered a larger total antioxidant capacity response across zones than the P treatment (S1 Table). The temperature treatment elicited zone-dependent increases in the total antioxidant capacity of estuarine anemones (Fig 5C; Table 1). In particular, the increase of 20°C caused a decrease in total antioxidant capacity only in the intertidal estuarine anemones (Table 1). Finally, subtidal estuarine anemones exposed to salinity of 30 ppt showed higher levels of total antioxidant capacity than those exposed to a reduced salinity of 10 ppt (Fig 5D). This effect was not observed for intertidal estuarine anemones, where a statistically significant interactive effect of salinity and zone on antioxidant production was found (Table 1; S1 Table). The model accounted for 71% of variation in total antioxidant capacity (R^2^ = 0.71).

## Discussion

The anthozoan *A*. *hermaphroditica* in an invertebrate that lives burrowed in the sediments of estuarine environments in southern Chile, where temporal changes in salinity, temperature, and UV–B exposure, influenced by tidal changes, can expose them to stressful conditions during different periods of time. It is especially stressful when low tide events occur during midday when temperature and radiation reach maximum daily levels [26]. In this study we estimated the cellular response of environmentally adapted anemones to different bathymetric zones through a multifactorial approach; significant differences in responses against experimental stressors were observed between estuarine anemones from intertidal and subtidal zones of the estuary. Our results indicate that adults of *A*. *hermaphroditica* from intertidal zones exhibited lower levels of oxidative damage and antioxidant responses than subtidal organisms when they were exposed experimentally to different combinations of salinity, temperature and UV-R.

### Environmental stressors and oxidative damage response

Exposure time, as well as reduction in salinity (10 ppt), high temperature (30°C) and exposure to UV-B (PAB) generated high levels of oxidative damage to the lipids in *A*. *hermaphroditica*. Baseline levels of lipid peroxidation observed in this study are within the range recorded for this organism in the Quempillén estuary during winter (25–60 nmol MDA g FW^-1^) [26]. Although in this study levels of lipid peroxidation were estimated based on the surrounding environmental conditions, a PCA analysis indicated that during winter salinity explained 76% of the cellular response in *A*. *hermaphroditica*; whilst during summer 98.73% of the cellular response was explained by temperature and UV-B radiation [26]. Although lower salinity increased lipid peroxidation in estuarine anemones, it did not affect protein carbonyl levels.

The environmental salinity in the Quempillén estuary fluctuates between 7 and 32 ppt [27], due to tidal changes as well as prolonged rainfall in the area, impacting the cellular, physiological and behavioural responses of *A*. *hermaphroditica*. Our results indicate that a reduced salinity of 10 ppt generated higher levels of lipid peroxidation in *A*. *hermaphroditica* compared to 30 ppt. Although the cellular response of anemones to salinity changes has received little attention, its effect on corals has been widely studied. For example, salt reduction from 33 to 20 ppt generated an increase in lipid peroxidation from 0.01 to 0.07 μmol MDA mg^-1^ total protein in the coral *Psammocora contigua* [33].

Sea anemones are osmoconformer invertebrates [34], therefore reduced salinity can generate a decrease in the concentration of osmotically active solutes in the internal medium as a mechanism that compensates for the external environment [35]. The above lies in the cell volume regulation, which is associated with changes of concentration of osmotically active substances (free amino acids; ninhydrin-positive substances, DMSP and K^+^) and also with modifications in membrane-bound transporters [35]. Storm-related reductions in salinity can generate high levels of cellular stress in tropical corals, which is manifested by the expulsion of 70% of symbiotic microalgae (bleaching) [33]. *Histological evidence of the effects of hyposaline exposure on the coral Stylophora pistillata demonstrated that degradation of the membranes, loss of zooxanthellae and tissue necrosis are expressions of oxidative damage in the tissues [36]. Transfer of the anemone Exaiptasia pallida from a saline condition of 30 ppt to hypersaline conditions (36 ppt) generated significant increases in the expression of HSP70, on the order of 6–11 times [37].* Thus the response of anthozoans to salinity fluctuations is associated with adaptive levels of each individual to the environmental conditions in which it develops.

Salinity fluctuations generate significant reductions in oxygen consumption rates in other anemones such as *Metridium senile*, mainly due to tentacular retraction and the generation of mucus on the body column as a strategy to isolate from the unfavourable external environment [38].

In this study, no significant differences in lipid peroxidation levels generated by the interaction of bathymetric zone and salinity can be argued, considering that intertidal and subtidal anemones were exposed directly to different levels of salinity after being removed from the sediment where they inhabit naturally. Although living in an estuary represents a permanent cellular and physiological challenge for *A*. *hermaphroditica* [26], living buried in sediment may minimize cell damage from hypo-osmotic conditions, a behavioural response also observed in other estuarine invertebrates such as *Cancer edwarsii* [2]. The role of sediment as a matrix to retain high salinity levels in estuarine ecosystems was evaluated under laboratory conditions, indicating that interstitial water can retain salinity up to 27.3 ppt, in contrast to salinity of 2 ppt observed in the water column [2]. Thus exposure to salinity levels either within (30 ppt) or outside (10 ppt) the physiological range of tolerance found in the sediment in the Quempillén estuary could not generate a difference in lipid peroxidation levels in *A*. *hermaphroditica*.

*Temperature is considered a major metabolic modulator of marine organisms, as high temperature induces high levels of oxidative damage, playing a pivotal role in the distribution of different marine organisms [39–41]*. Although the high temperature (30°C) used in this study increased levels of lipid peroxidation in *A*. *hermaphroditica*, exposure time is essential in the oxidative response of *A*. *hermaphroditica*. Similar levels of lipid peroxidation between intertidal and subtidal organisms exposed for a period of over 24 h at 30°C indicated that high temperatures generate a deleterious effect which can cause irreversible damage to the cell, triggering animal death. This has been confirmed in the symbiotic anemone *Aiptasia pallida*, where exposure to prolonged periods of high temperature generated a significant increase in apoptotic activity (programmed cell death / PCD) and cell necrosis as a result of irreversible oxidative damage [42].

*Environmental records of the Quempillén estuary indicate that anemones are exposed to water temperatures ranging between 9–30.2°C during the year [26, 27]. Our results showed higher levels of lipid peroxidation in A. hermaphroditica from the subtidal zone, which could be associated with overproduction of ROS in the mitochondria at experimental temperatures of 30°C, which exceeds the thermal range to which subtidal anemones are normally exposed. A temperature increase of up to 30°C has a direct effect on biological membranes, whose main components are the double bonds of polyunsaturated fatty acids* (PUFAs) [43], which generates elevated levels of lipid peroxidation as was observed in this experiment. It is well known that increases in temperature in the water column decrease dissolved oxygen [44], which requires increases in the respiratory capacities of animals to meet the oxygen demands necessary for the generation of ATP [45]. This leads to rapid accumulation of cellular ROS, which is a direct reflection of oxygen consumption [45]. According to previous exposures of *A*. *hermaphroditica* to temperatures of 30°C for >48 h, where high mortality was observed (V. Cubillos, pers. obs.), it was evident that the maximal exposure time of our experiment exceeded that to which the animals are usually exposed in nature. Thus subtidal anemones may *generate increased levels of oxidative damage in an attempt to match their physiology to the changing environment*.

Independently of the bathymetric zone, radiation treatments had the same deleterious cellular effects in anemones from intertidal and subtidal areas. However, UV-B (PAB radiation treatment) induced higher lipid peroxidation levels in *A*. *hermaphroditica* than PAR (P radiation treatment) or UV-A (PA radiation treatment) treatments, mainly since its indirect absorption can generate overproduction of cell ROS [19, 46]. UV-B induced lipid peroxidation levels in this study (baseline: 19–28 nmol mg FW^-1^; and final: 50–57 nmol mg FW^-1^) are higher than those reported for the sea anemone *Actinia tenebrosa* under similar experimental conditions [47].

During periods of low tide in the Quempillén estuary, subtidal organisms can be submerged 20 to 30 cm below the surface. This would not generate major differences in the levels of photo-oxidative damage caused by the attenuation of UV-B in the water column between the bathymetric zones, which would explain the results obtained in this study. UV-B has an attenuation coefficient that allows it to penetrate thorough the water column up to 20 meters deep [48], which could generate high levels of cell damage in anemones in the Quempillén estuary, considering that they inhabit shallow environments.

The symbiont condition (zooxanthellae) of *A*. *hermaphroditica* [49] can generate an excessive ROS production (^1^O_2_, O_2_^●-^ y H_2_O_2_) [50, 51] as a result of UV-B exposure over environmental levels, which can impact protein synthesis, membrane integrity and HO^●^ generation [52, 53]. For symbiont cnidarians, the combined effects of high radiation and high temperatures can generate a break in the equilibrium between the host and the zooxanthellae since these factors usually impact the expulsion of the symbionts [52–55]. Consequently, the exposure of symbiont microalgae to high levels of temperature and UV-B radiation would generate an overproduction of O_2_^●—^and H_2_O_2_, which additionally generates a reduction in the quantum yield of PSII fluorescence, affecting ribulose-1,5-bisphosphate carboxylase/oxygenase (RUBISCO) activity [56].

Protein carbonylation levels in *A*. *hermaphroditica* were within the levels observed for the species in the natural environment [26]. UV-B (PAB radiation treatment) induced carbonyl groups in this study (4–5.5 μmol carbonyl mg protein^-1^) were in the range reported for the intertidal anemone *A*. *tenebrosa* (3–10.5 μmol carbonyl mg protein^-1^) exposed to a similar experimental radiation condition [47]. Exposure time, type of radiation treatment and temperature significantly induce protein carbonyl level in *A*. *hermaphroditica*, considering not only the noxious effect of UV-B, but also that the level depends upon the exposure time [47]. Salinity and bathymetric zone alone did not influence the generation of carbonyl groups in this experiment. This is associated with the fact that carbonyl groups are formed rapidly in proteins as a cellular response to stress, with high stability over time [57]. Thus the difference in experimental salinity (10 and 30 ppt) could possibly have had an impact on the generation of carbonyls, but since the oxidative damage estimation was carried out every 24 h, the period was too long to be able to determine potential differences in carbonyl production between intertidal and subtidal anemones. It is important to highlight that protein carbonyls are also generated as a by-product of lipid peroxidation [57], which may indicate that lipid peroxidation in *A*. *hermaphroditica* might contribute to the generation of carbonyl groups under different abiotic treatments.

### Antioxidant capacity against environmental stress in *A*. *hermaphroditica*

According to our results, subtidal anemones are more susceptible to induce lipid peroxidation in response to environmental fluctuations than intertidal anemones, which triggers the synthesis of increased levels of antioxidants. This result is comparable to that of *Mytilus galloprovincialis*, in which subtidal mussels exposed to air increased SOD and CAT antioxidant activity by 2 and 3-fold, respectively, compared to intertidal organisms kept under the same condition [58]. Since the estuary of the Quempillén River is small and located in a mid-latitude zone [27], changes in environmental conditions can amplify the cellular effect in *A*. *hermaphroditica* in a short time period. Lower total antioxidant capacity levels in intertidal than subtidal anemones allow us to infer that the former can anticipate an ROS insult as a mechanism to prepare for an oxidative stress event [59, 60]. A metagenomics study indicated that intertidal communities develop more complex stress tolerance systems than subtidal ones, which is based on the use of antioxidant mechanisms, secondary metabolites and adaptation to environmental conditions [61]. The use of secondary metabolites such as mycosporine-like amino acids (MAAs) is a widely observed strategy in this type of anemone [62–64], both for its photoprotective role and for its antioxidant capabilities [43, 65, 66].

The compound mycosporine 2-glycine (λ_max_: 331 nm) has been identified in *A*. *hermaphroditica*; its concentration is maintained year round in the Quempillén estuary [26]. The ability to upregulate stress-response genes in relation to a stressor factor may determine the physiological response of an organism. Specific adaptations of sea anemones to acute and chronic stress periods have been related to heat shock proteins (HSP) that maintain the cellular response through the refolding of proteins because of oxidative damage [67, 68]. Although we did not estimate HSP in *A*. *hermaphroditica*, we may infer that an efficient mechanism of protein repair may minimize the effect of environmental fluctuations on oxidative damage. Genetic components that regulate the synthesis of hypoxia-induced factor (HIF), receptors, signal transduction and transcription factors are described as pivotal mechanisms involved in the “chemical defensome” of sea anemones against environmental stress [69].

## Conclusions

The present study demonstrates that the estuarine anemone *A*. *hermaphroditica* responds differently to sudden changes in environmental fluctuations at the Quempillén estuary according to its bathymetric origin. This is produced by a process of local acclimation to subtidal and intertidal environmental conditions where salinity, temperature and UV-B levels can generate different microenvironments during low tide. Subtidal anemones exhibited higher cell damage levels and total antioxidant capacity than their intertidal counterparts after abiotic stress. This can be associated with the fact that their bathymetric zone is continuously underwater, exposing them to more stable levels than intertidal anemones. IPCC predicts future increases in the frequency and intensity of rainfall in mid-latitude zones, impacting salinity levels in coastal areas [70]. Compared to subtidal organisms, intertidal organisms may have greater resistance and resilience to environmental conditions in a climate change scenario, due to a larger range of physiological tolerance. Future studies should focus on understanding the physiological responses of coastal organisms to stochastic events such as heat waves, which can generate high levels of cell stress in those animals not acclimatized to extreme environmental conditions.

## Supporting information

S1 Table Post-hoc analyses for lipid peroxidation, protein carbonyl levels, and antioxidant capacity in sea anemones exposed to experimental radiation (*R*, three levels: P, PA, or PAB), temperature (*Te*, two levels: 10° or 30°C), and salinity (*S*, two levels: 10 or 30 PSU) treatments. The experiments were conducted for sea anemones from two bathymetric zones (intertidal or subtidal) for 48 hrs (measurements done at 0, 24, or 48 hrs). For each response variable, the global model took the form of *y* = Z*(*T*+*R*+*Te*+*S*). In the instance of statistically significant interactive effects, pairwise comparisons were conducted separately for each zone. In the case of main effects, the pairwise comparisons were conducted after pooling both zones (i.e., “Both” in the Zone column). P-values < 0.05 are in **bold.**
(DOCX)
