# Supplementary material for: Specific plasticity of the anemone Anthopleura hermaphroditica to intertidal and subtidal environmental conditions of the Quempillén estuary
Source: PLoS One. 2023 Jan 5;18(1):e0279482. doi: 10.1371/journal.pone.0279482 (PMC9815623; doi:10.1371/journal.pone.0279482)
Supplement: S1 Table — The experiments were conducted for sea anemones from two bathymetric zones (intertidal or subtidal) for 48 hrs (measurements done at 0, 24, or 48 hrs). For each response variable, the global model took the form of y = Z*(T+R+Te+S). In the instance of statistically significant interactive effects, pairwise comparisons were conducted separately for each zone. In the case of main effects, the pairwise comparisons were conducted after pooling both zones (i.e., “Both” in the Zone column). P-values < 0.05 are in bold. (DOCX) [file pone.0279482.s001.docx]

S1 Table. Post-hoc analyses for lipid peroxidation, protein carbonyl levels, and antioxidant capacity in sea anemones exposed to experimental radiation (*R,* three levels: P, PA, or PAB), temperature (*Te,* two levels: 10º or 30º C), and salinity (*S,* two levels: 10 or 30 PSU) treatments. The experiments were conducted for sea anemones from two bathymetric zones (intertidal or subtidal) for 48 hrs (measurements done at 0, 24, or 48 hrs). For each response variable, the global model took the form of $y=Z*(T+R+Te+S)$. In the instance of statistically significant interactive effects, pairwise comparisons were conducted separately for each zone. In the case of main effects, the pairwise comparisons were conducted after pooling both zones (i.e., “Both” in the Zone column). P-values < 0.05 are in **bold.**

| Response | Factor | Contrast | Zone | Estimate | SE | t | P |
| --- | --- | --- | --- | --- | --- | --- | --- |
| **Lipid peroxidation** | Time | 24 - 0 | Intertidal | 19.31 | 4.89 | 3.95 | **<0.001** |
|  |  | 48 - 0 |  | 55.21 | 4.89 | 11.30 | **<0.001** |
|  |  | 48 - 24 |  | 35.91 | 4.89 | 7.35 | **<0.001** |
|  |  | 24 - 0 | Subtidal | 45.11 | 4.89 | 9.23 | **<0.001** |
|  |  | 48 - 0 |  | 48.85 | 4.89 | 10.00 | **<0.001** |
|  |  | 48 - 24 |  | 3.74 | 4.89 | 0.77 | 0.724 |
|  | Radiation | PA - P | Both | 8.12 | 3.45 | 2.35 | 0.051 |
|  |  | PAB - P |  | 31.08 | 3.45 | 9.00 | **<0.001** |
|  |  | PAB - PA |  | 22.96 | 3.45 | 6.65 | **<0.001** |
|  | Temperature | 30 - 10 | Intertidal | 10.28 | 3.99 | 2.58 | **0.010** |
|  |  | 30 - 10 | Subtidal | 25.32 | 3.99 | 6.35 | **<0.001** |
|  | Salinity | 10 - 30 | Both | 14.87 | 2.82 | 5.27 | **<0.001** |
| **Protein carbonyls** | Time | 24 - 0 | Intertidal | 3.67 | 0.32 | 11.36 | **<0.001** |
|  |  | 48 - 0 |  | 8.23 | 0.32 | 25.51 | **<0.001** |
|  |  | 48 - 24 |  | 4.57 | 0.32 | 14.15 | **<0.001** |
|  |  | 24 - 0 | Subtidal | 2.71 | 0.32 | 8.39 | **<0.001** |
|  |  | 48 - 0 |  | 3.68 | 0.32 | 11.39 | **<0.001** |
|  |  | 48 - 24 |  | 0.97 | 0.32 | 3.00 | **0.008** |
|  | Radiation | PA - P | Both | 0.34 | 0.23 | 1.51 | 0.289 |
|  |  | PAB - P |  | 0.92 | 0.23 | 4.04 | **<0.001** |
|  |  | PAB - PA |  | 0.58 | 0.23 | 2.54 | **0.031** |
|  | Temperature | 30 - 10 | Intertidal | 1.05 | 0.26 | 3.97 | **<0.001** |
|  |  | 30 - 10 | Subtidal | -0.09 | 0.26 | -0.33 | 0.739 |
| **Antioxidant capacity** | Time | 24 - 0 | Intertidal | 27.51 | 1.94 | 14.19 | **<0.001** |
|  |  | 48 - 0 |  | 30.43 | 1.94 | 15.69 | **<0.001** |
|  |  | 48 - 24 |  | 2.93 | 1.94 | 1.51 | 0.288 |
|  |  | 24 - 0 | Subtidal | 6.88 | 1.94 | 3.55 | **0.001** |
|  |  | 48 - 0 |  | 1.16 | 1.94 | 0.60 | 0.822 |
|  |  | 48 - 24 |  | -5.73 | 1.94 | -2.95 | **0.010** |
|  | Radiation | PA - P | Both | 2.23 | 1.37 | 1.63 | 0.235 |
|  |  | PAB - P |  | 4.79 | 1.37 | 3.49 | **0.002** |
|  |  | PAB - PA |  | 2.55 | 1.37 | 1.86 | 0.151 |
|  | Temperature | 30 - 10 | Intertidal | -4.96 | 1.58 | -3.13 | **0.002** |
|  |  | 30 - 10 | Subtidal | 0.01 | 1.58 | 0.00 | 0.997 |
|  | Salinity | 30 - 10 | Intertidal | -3.06 | 1.58 | -1.93 | 0.054 |
|  |  | 30 - 10 | Subtidal | 3.66 | 1.58 | 2.31 | **0.022** |
